# Anti-*Campylobacter* Activities and Resistance Mechanisms of Natural Phenolic Compounds in *Campylobacter*

**DOI:** 10.1371/journal.pone.0051800

**Published:** 2012-12-17

**Authors:** Anja Klančnik, Sonja Smole Možina, Qijing Zhang

**Affiliations:** 1 Department of Food Science and Technology, Biotechnical Faculty, University of Ljubljana, Ljubljana, Slovenia; 2 Department of Veterinary Microbiology and Preventive Medicine, Iowa State University, Ames, Iowa, United States of America; Royal College of Surgeons, Ireland

## Abstract

**Background:**

*Campylobacter* is a major foodborne pathogen and alternative antimicrobials are needed to prevent or decrease *Campylobacter* contamination in foods or food producing animals. The objectives of this study are to define the anti-*Campylobacter* activities of natural phenolic compounds of plant origin and to determine the roles of bacterial drug efflux systems in the resistance to these natural phenolics in *Campylobacter jejuni*.

**Methodology/Principal Findings:**

Anti-*Campylobacter* activities were evaluated by an MIC assay using microdilution coupled with ATP measurement. Mutants of the *cmeB* and *cmeF* efflux genes and the *cmeR* transcriptional repressor gene were compared with the wild-type strain for their susceptibilities to phenolics in the absence and presence of efflux-pump inhibitors (EPIs). The phenolic compounds produced significant, but variable activities against both antibiotic-susceptible and antibiotic resistant *Campylobacter*. The highest anti-*Campylobacter* activity was seen with carnosic and rosmarinic acids in their pure forms or in enriched plant extracts. Inactivation of *cmeB* rendered *C. jejuni* significantly more susceptible to the phenolic compounds, while mutation of *cmeF* or *cmeR* only produced a moderate effect on the MICs. Consistent with the results from the efflux pump mutants, EPIs, especially phenylalanine-arginine β-naphthylamide and NMP, significantly reduced the MICs of the tested phenolic compounds. Further reduction of MICs by the EPIs was also observed in the *cmeB* and *cmeF* mutants, suggesting that other efflux systems are also involved in *Campylobacter* resistance to phenolic compounds.

**Conclusion/Significance:**

Natural phenolic compounds of plant origin have good anti-*Campylobacter* activities and can be further developed for potential use in controlling *Campylobacter*. The drug efflux systems in *Campylobacter* contribute significantly to its resistance to the phenolics and EPIs potentiate the anti-*Campylobacter* activities of plant phenolic compounds.

## Introduction

*Campylobacter jejuni* is the leading bacterial cause of human food-borne enteritis in many industrialised countries. Food-borne exposure to *Campylobacter* spp. is most frequent through consumption of undercooked, contaminated broiler chicken meat, and through cross-contamination with other foods during meat preparation [1]. Additionally, *Campylobacter* spp. have become increasingly resistant to antimicrobials, which thus compromises the effectiveness of its control in the food chain as well as antibiotic treatments [2], [3].

The control of *Campylobacter* spp. represents a major goal for the improvement of food safety and public health. Different types of alternative bioactive compounds have been screened for potential anti-*Camyplobacter* effects. A potential strategy for controlling foodborne pathogens, including *Campylobacter*, is screening, development and use of natural antimicrobial and resistance-modifying agents, preferably derived from plants because of their Generally Recognised as Safe (GRAS) status [4].

Plants are known to produce an enormous variety of the small-molecule antibiotics that are generally classified as ‘phytoalexins’. The structural molecular space of these phytoalexins is diverse, as they include terpenoids, glycosteroids, flavonoids and polyphenols. They generally have weak antibiotic activities that are several orders of magnitudes less than those of the common antibiotics produced by bacteria and fungi [5]. However, although such plant-derived antibacterials are less potent, plants can fight off infections successfully [5] and plant-based antibacterials can be further modified to enhance efficacy.

Among others, phenolic extracts from many different plant materials have been characterized [6]–[9]. As an example, rosemary (*Rosemarinus officinalis* L.) is an aromatic plant that has been successfully exploited for commercial use as an antioxidant and antimicrobial, and its extracts are widely used in cosmetic and pharmacetucial products and in the food [10].

Other examples are grape skin and vine leaf extracts of *Vitis vinifera* varieties [11]. These extracts are of increasing interest in the food industry because they reduce the oxidative degradation of lipids and can thereby improve the quality and nutritional value of foods [12], [13]. Additionally, these extracts have antimicrobial activities. The sensitivity of bacteria to polyphenols depends on the bacterial species and the structure of the polyphenol [14], [15]. *Campylobacter* spp., different from other food-borne bacteria, have unique surface structures and lack the typical stress-adaptive responses [16], [17]. In general, campylobacters are more sensitive to different phenolics than other enteric pathogens [4], [18].

Multiple mechanisms associated with antibiotic resistance have been identified in *Campylobacter* spp., including target mutations, antibiotic modification/inactivation, and drug efflux [2], [19]. The main RND (resistance-nodulation-cell division)-type efflux pump, known as CmeABC, mediates the extrusion of structurally diverse antimicrobials and contributes to intrinsic and acquired resistance to various antimicrobials [20]–[22]. This system is encoded by a three-gene operon and is composed of a transporter protein (CmeB), a periplasmic membrane fusion protein (CmeA), and an outer membrane factor (CmeC). Expression of *cmeABC* is regulated by CmeR, a transcription repressor that is encoded by a gene immediately upstream of *cmeA* [23], [24]. CmeR binds directly to an inverted repeat in the promoter region of *cmeABC* and inhibits the transcription of this efflux operon [23], [25]. In addition, *C. jejuni* has another RND-type efflux system, CmeDEF, which plays a secondary role in conferring intrinsic resistance, with CmeD, CmeE and CmeF as an outer membrane channel protein, periplasmic fusion protein and inner membrane transporter, respectively. CmeDEF has different substrate-binding properties and interacts with CmeABC in conferring antimicrobial resistance [26].

The goal of this study is to evaluate the anti-*Campylobacter* activities of various plant phenolics and assess if efflux mechanisms are involved in the resistance of *C. jejuni* to these phenolics (pure phenolic compounds and extracts of plant phenolics). First, we analyzed the susceptibilities of *C. jejuni* isolates of various origins, wild-type *C. jejuni* 11168 and its efflux mutants (*cmeB, cmeF* and *cmeR*) to these phenolic compounds. Second, we used known efflux-pump inhibitors (EPIs) to determine if the EPIs potentiate the anti-*Campylobacter* activities of the natural phenolic compounds. Our findings demonstrate the potential use of plant-based phenolics in controlling *Campylobacter* and provide new insights into the resistance mechanisms of *Campylobacter* to the antimicrobials of plant origin.

## Materials and Methods

### Bacterial Strains, Generation of Efflux Mutants, and Growth Conditions

Eleven food, animal, water and human *Campylobacter* strains were used in the present study. They were isolated and identified phenotypically and by multiplex polymerase chain reaction (mPCR), as described previously [27]. The reference human clinical isolate of *C. jejuni* NCTC 11168 was provided by Sophie Payot (French National Institute for Agricultural Research, UR086 BioAgresseurs, Santè e Environnement, Nouzilly, France). Natural transformation [28] was used to generate the mutants of *cmeB*, *cmeF*, and *cmeR*. In the transformation experiment, the donor DNA was genomic DNA prepared from the corresponding mutant strains published previously [20], [23], [26] and the recipient strain was NCTC 11168. The transformants of *cmeB* (referred to as 11168B) were selected on Müller Hinton (MH) agar (Oxoid, Hampsire, UK) with 30 µg kanamycin/mL, while the *cmeF* (11168F) and *cmeR* (11168R) transformants were selected on MH agar plates with 4 µg chloramphenicol/mL. The mutants of *cmeB, cmeF* and *cmeR* were confirmed by PCR using specific primers (Table 1). The cultures were stored at −80°C in brain–heart infusion broth (Oxoid) supplemented with 5% horse blood (Oxoid) and glycerol (Kemika, Zagreb, Croatia). The isolates were sub-cultured on Columbia agar (Oxoid) supplemented with 5% horse blood (Oxoid), at 42°C in gas-tight containers under micro-aerobic conditions (5% O_2_, 10% CO_2_, 85% N_2_).

**Table 1:** PCR primer pairs used in the present study.

Target gene	Primer pair	*n*-mer	Sequence (5′–3′)	Reference or source
*cmeB*	cmeB BF1	24	GCT GGA TCC ATA GGT CTT ACA AAT	Lin et al., 2002 [20]
	cmeB CR	27	TTT TTA AAG CTT TAA GGT AAT TTT CTT	Lin et al., 2002 [20]
*cmeF*	cmeF FF1	24	AAG TAC AAC TCT CAT TGC TTG CAT	Akiba et al., 2006 [26]
	cmeF FR1	20	TGG CTA TTG CCA TAG GAG AA	Akiba et al., 2006 [26]
*cmeR*	cmeR F	24	TAG AAA AGT ATA TTT GTA TAC CCT	Lin et al., 2005a [23]
	cmeR GSR4	21	GAA ATTT TTG GCT AAT TATAT	Lin et al., 2005a [23]

### Pure Phenolic Compounds and Extracts of Plant Phenolics

The natural phenolic compounds used in the present study included nine pure phenolic compounds and 22 extracts of plant phenolics. The pure phenolic compounds were: (−)-epigallocatechin gallate (EGCG), chlorogenic acid, gallic acid, sinapinic acid, vanillic acid, syringic acid, ferulic acid (all from Sigma-Aldrich GmbH, Steinheim, Germany), rosmarinic acid and carnosic acid (both from Chromadex, Santa Ana, CA, USA). The extracts of plant phenolics used included commercially available rosemary (*Rosemarinus officinalis* L) extracts with different contents of carnosic acid (CA) and rosmarinic acid (RA): I18 (18.8% CA), V40 (40% CA), V70 (70% CA), A40 (40% RA) (Vitiva, Markovci, Slovenia). The other extracts were prepared from sage (*Salvia officinalis)*, peppermint (*M. balsamea* Willd), lemon balm (*Melissa officinalis*), oregano (*Origanum vulgare*), green tea (*Camellia sinensis*), thyme (*Thymus mongolicus*), bearberry *(Arctostaphylos uva ursi*), black seeds (*Nigella sativa*) as well as from grapes skin and leaf extracts of *Vitis vinifera* L. from different red (*Lasin, Merlot, Vranac, Babić*) and white (*Rkaciteli, Zlatarica*, *Debit, Kujundžuša, Trnjak, Rudežuša*) grape varieties as described previously [4], [11], [13], [29].

Briefly, plant phenolic extracts were lyophilised and then dissolved in absolute ethanol to provide the stock solutions. They were further diluted in the appropriate media to the working concentrations. Two-fold serial dilutions of the pure phenolic compounds and the herb were used at concentrations from 0.6 µg/mL to 1,250 µg/mL, as for all of the vine leaf and grape skin extracts at concentrations from 7.8 µg/mL to 16,000 µg/mL.

### PCR Confirmation of the Gene Knock-out Mutants

The genomic DNA was extracted using the PrepMan Ultra sample preparation reagent (Applied Biosystems, Foster City, California, USA) from pure cultures of the wild-type NCTC 11168 and its mutant strains grown in Müller Hinton broth (Oxoid). One mL of overnight culture was centrifuged at 13,000× *g* for 3 min to pellet the bacteria. The pellet was resuspended in 100 µL PrepMan Ultra sample preparation reagent, mixed for 30 s, and heated in a water-bath at 95°C for 10 min. The suspension was again centrifuged at 13,000× *g* for 3 min, and the supernatant was removed into a fresh tube. The PCR primers used in the present study and the expected sizes of the products are listed in Table 1. The PCR mix and the cycling conditions varied according to the expected sizes of the products. PCR amplifications for *cmeF* and *cmeR* were performed in a 25-µL reaction volume containing 10× RED Taq PCR buffer, 25 mM MgCl_2_, 20 mM dNTP (Promega, Madison, USA), 300 nM forward primer and 300 nM reverse primer (Table 1), 1 U/µL RED Taq polymerase (Sigma-Aldrich GmbH, Steinheim, Germany) and 2 µL DNA lysate. The PCR was performed in a 2400 GeneAmp thermal cycler PCR system (Perkin Elmer, Waltham, Massachusetts, USA) at 95°C for 300 s (one cycle), 95°C for 15 s, 50°C for 30 s, and 72°C for 45 s (35 cycles); plus 72°C for 7 min (one cycle). PCR amplification for *cmeB* was performed in a 20-µl reaction volume containing 5× Phusion High-Fidelity DNA polymerase buffer (New England Biolabs, Herts, UK), 25 mM MgCl_2_, 20 mM dNTP, 300 nM forward primer and 300 nM reverse primer (Table 1), 1 U/µL Phusion High-Fidelity DNA polymerase (New England Biolabs, Herts, UK) and 2 µL DNA lysate. The cycling conditions for the PCR were at 98°C for 30 s (one cycle); 98°C for 10 s, 50°C for 30 s, and 72°C for 60 s (30 cycles); plus 72°C for 7 min (one cycle). The PCR products were electrophoresed on 2% agarose gels.

### Antimicrobial Susceptibility Testing

The broth microdilution method was used for measuring the MICs as described previously [4]. The MICs were defined as the lowest concentration of an antimicrobial where no metabolic activity is seen after 24 h, and they were determined on the basis of the bioluminescence signal measured using a microplate reader (Tecan, Mannedorf/Zurich, Switzerland) after adding the CellTiter-Glo reagent (Promega Corporation, Madison, USA) to the culture media [4]. All of the MIC measurements were carried out in duplicate or triplicate. The control wells were prepared with culture medium, with the bacterial suspension only, or alternatively with the antimicrobial only, and with ethanol corresponding to the highest concentration present in the preparations. The ethanol controls did not show any inhibitory effects on the growth of the strains tested (data not shown).

### Efflux Pump Inhibitors

To investigate the contributions of antibiotic efflux pumps in natural antimicrobial resistance, the wild-type and mutant strains were tested with the phenolic compounds in the absence and presence of EPIs. The MICs of the tested wild-type and *cmeB, cmeR* and *cmeF* mutants were determined using the broth microdilution method in the absence and presence of five EPIs: PAβN, NMP (Chess, Mannheim, Germany), verapamil, reserpine and CCCP (Sigma-Aldrich). For this purpose, Müller Hinton broth was supplemented with 20 µg/mL PAβN, 100 µg/mL NMP, 100 µg/mL verapamil, 100 µg/mL reserpine or 0.25 µg/mL CCCP. Microdilution tests were also performed in preliminary independent experiments to determine the MICs of the EPIs used for all of the strains tested. The selected concentrations of the EPIs had no inhibitory effects on bacterial growth for any of the strains tested.

### Statistical Analysis

The MICs shown in Table 2 were compared using the independent-samples T-tests to define the significance of the differences in resistances between *C. jejuni* and *C. coli*, between erythromycin-susceptible and erythromycin-resistant isolates, and between pure phenolic compounds and phenolic extracts. For the data in Tables 3, 4, and 5, the fold differences in MICs were log2 transformed and were used for statistical analyses. One sample *t* test was used to test the null hypothesis that there was no difference [log_2_(fold difference) = 0] in the MICs between the wild type strain and a mutant strain (Table 3) or between EPI-treated and non-treated in a given strain (Tables 4 and 5). Results were considered significant when *P*≤0.05. Statistical analyses were performed with IBM SPSS statistic software, v18.0.

**Table 2:** Susceptibilities of *Campylobacter* spp. and strains of various origins to pure phenolic compounds and phenolic extracts of plant origin*.

*Campylobacter strain*	Source	Carnosic acid	Rosmarinic acid	Chlorogenic acid	Syringic acid	Ferulic acid	V40	V70	A40	Sage	Peppermint	Oregano	Green tea	Babić	Merlot	Zlatarica
		MIC (µg/mL)														
**Erythromycin resistant**																
*C. coli* 137	Poultry	19.5	156	313	313	78	78	78	156	156	156	625	156	8,000	1,000	1,000
*C. coli* 140	Poultry	39	78	156	156	313	78	78	625	156	313	625	156	8,000	1,000	1,000
*C. coli* 171	Poultry	19.5	156	156	156	313	78	78	156	156	313	625	156	4,000	4,000	4,000
*C. coli* FC8	Poultry	39	156	313	313	313	78	39	625	313	313	625	313	4,000	500	1,000
*C. coli* FC10	Poultry	39	78	313	313	78	78	78	313	313	156	625	313	4,000	2,000	2,000
*C. coli* VC7114	Pig	39	78	313	313	78	78	78	156	313	156	1250	313	8,000	1,000	2,000
*C. coli* VC11076	Pig	78	156	156	156	156	78	78	156	313	156	1250	313	4,000	4,000	4,000
*C. jejuni* 375-06	Human	78	156	313	156	156	78	156	313	156	313	1250	156	8,000	2,000	2,000
**Erythromycin susceptible**																
*C. jejuni* K49/4	Poultry	19.5	78	313	313	313	156	156	156	156	156	1250	156	8,000	1,000	1,000
*C. jejuni* 807	Water	39	156	156	313	313	156	78	313	156	156	625	156	4,000	1,000	2,000
*C. jejuni* 573/03	Human	39	156	156	313	156	156	78	625	156	156	1250	156	4,000	2,000	2,000

* The MICs of various antibiotics in the examined isolates are reported in references [30], [35].

**Table 3:** Susceptibilities of *C. jejuni* 11168 and its efflux mutants to pure phenolic compounds and phenolic extracts of plant origin.

Antimicrobial	11168	11168B*			11168F		11168R		
	MIC (µg/mL)	MIC (µg/mL)		Fold diff.	MIC (µg/mL)	Fold diff.	MIC (µg/mL)	Fold diff.	
**Phenolic compounds**									
EGCG	78	78	1		78	1	313	**0.25**	
Rosmarinic	156	1.2	**128**		313	0.5	313	0.5	
Carnosic	19.5	19.5	1		39	0.5	78	**0.25**	
Chlorogenic	313	4.9	**64**		313	1	313	1	
Gallic	313	4.9	**64**		78	**4**	78	**4**	
Sinapinic	313	78	**4**		156	2	156	2	
Vanillic	313	39	**8**		313	1	156	2	
Syringic	313	78	**4**		156	2	156	2	
Ferulic	313	78	**4**		156	2	156	2	
**Rosemary extracts**									
I18	313	19.5	**16**		625	0.50	156	2	
V40	78	9.8	**8**		156	0.50	156	0.50	
V70	78	4.9	**16**		78	1	78	1	
A40	156	2.4	**64**		156	1	313	0.5	
**Herb extracts**									
Sage	313	4.9	**64**		156	2	156	2	
Peppermint	156	9.8	**16**		156	1	156	1	
Lemon balm	625	9.8	**64**		156	**4**	313	2	
Oregano	1250	19.5	**64**		156	**8**	313	**4**	
Green tea	156	9.8	**16**		78	2	78	2	
Thyme	625	9.8	**64**		156	**4**	156	**4**	
Bearberry	313	2.4	**128**		1,000	**0.25**	1,000	**0.25**	
Black seeds	500	62.5	**8**		1,000	0.5	2,000	**0.25**	
**Grape leaf extracts**									
Lasin	1,000	62.5	**16**		1,000	1	2,000	0.5	
Merlot	1,000	62.5	**16**		1,000	1	2,000	0.5	
Vranac	500	62.5	**8**		1,000	0.5	1,000	0.5	
Babić	8,000	4,000	2		8,000	1	8,000	1	
Debit	500	62.5	**8**		500	1	1,000	0.5	
Zlatarica	1,000	31.3	**32**		500	2	500	2	
Kujundžuša	4,000	62.5	**64**		2,000	2	2,000	2	
Rkaciteli	4,000	62.5	**64**		2,000	2	2,000	2	
Trnjak	2,000	500	**4**		500	**4**	1,000	2	
Rudežuša	2,000	62.5	**32**		1,000	2	1,000	2	

“**Fold diff**” depicts fold difference, which is calculated using the formula: MIC of 11168/MIC of a mutant strain. ≥4-fold changes are indicated in bold.

* The MICs of 11168B are significantly lower than those of 11168 with phenolic compounds (*P<*0.05), rosemary extracts (*P<*0.01), herb extracts (*P<*0.01), and grape leaf extracts (*P<*0.01).

## Results and Discussion

### Anti-Campylobacter Activity of the Different Natural Phenolic Compounds

In previous studies of the antimicrobial activities of phenolic extracts from different plant sources, Klančnik et al. [4], [29] reported that campylobacters were more sensitive to different phenolic compounds or extracts than other examined enteric organisms, despite the fact that *Campylobacter* is a gram-negative bacterium. In the present study, we conducted a comprehensive evaluation of the anti-*Campylobacter* activities of the pure phenolic compounds and different plant extracts using *Campylobacter* isolates from different sources, various mutant constructs and EPIs.

**Table 4:** Susceptibilities of *C. jejuni* 11168 and its efflux mutants to phenolic compounds in the absence and presence of PAβN (20 µg/mL)^a^, NMP (100 µg/mL)^a^, verapamil (100 µg/mL), reserpine (100 µg/mL) or CCCP (0.25 µg/mL)^b^.

Phenolic acid orcompound ±inhibitor	11168		11168B		11168F		11168R	
	MIC (µg/mL)	Fold diff.	MIC (µg/mL)	Fold diff.	MIC (µg/mL)	Fold diff.	MIC (µg/mL)	Fold diff.
**EGCG**	78		78		78		313	
+PAβN	9.8	**8**	19.5	**4**	19.5	**4**	9.8	**32**
+NMP	78	1	19.5	**4**	19.5	**4**	9.8	**256**
+Verapamil	78	1	78.5	1	78	1	78	**4**
+Reserpine	78	1	78.5	1	78	1	78	**4**
+CCCP	19.5	**4**	156	0.5	9.8	**8**	19.5	**16**
**Rosmarinic**	156		1.2		313		313	
+PAβN	39	**4**	0.3	**4**	156	2	78	**4**
+NMP	39	**4**	1.2	1	78	**4**	156	2
+Verapamil	156	1	0.6	2	156	2	313	1
+Reserpine	156	1	0.6	2	78	**4**	313	1
+CCCP	19.5	**8**	0.6	2	39	**8**	156	2
**Carnosic**	19.5		19.5		39		78	
+PAβN	<0.6	**>32**	0.3	**64**	2.4	**16**	<0.6	**>128**
+NMP	4.9	**4**	2.4	**8**	39	1	39	2
+Verapamil	9.8	2	9.8	2	39	1	78	1
+Reserpine	9.8	2	19.5	1	39	1	78	1
+CCCP	4.9	**4**	19.5	1	39	1	78	1
**Chlorogenic**	313		4.9		313		313	
+PAβN	2.4	**128**	0.3	**16**	313	1	39	**8**
+NMP	625	0.5	2.4	2	625	0.5	78	**4**
+Verapamil	625	0.5	9.8	0.5	313	1	313	1
+Reserpine	625	0.5	4.9	1	625	0.5	625	0.5
+CCCP	78	**4**	1.2	**4**	156	2	156	2
**Gallic**	313		4.9		78		78	
+PAβN	<9.8	**>32**	0.3	**16**	19.5	**4**	<9.8	**>8**
+NMP	19.5	**16**	0.3	**16**	39	2	39	2
+Verapamil	78	**4**	4.9	1	39	2	78	1
+Reserpine	156	2	4.9	1	39	2	78	1
+CCCP	78	**4**	<0.3	**>16**	78	1	78	1
**Sinapinic**	313		78		156		156	
+PAβN	<9.8	**>32**	<1.2	**>64**	39	4	<9.8	**>16**
+NMP	<9.8	**>32**	<1.2	**>64**	78	**2**	156	1
+Verapamil	39	**8**	156	1	78	2	156	1
+Reserpine	19.5	**16**	78	1	78	2	156	1
+CCCP	78	**4**	<1.2	**>64**	156	1	156	1
**Vanillic**	313		39		313		156	
+PAβN	<0.6	**>512**	9.8	**4**	156	2	9.8	**16**
+NMP	4.9	**64**	2.4	**16**	156	2	78	2
+Verapamil	78	**4**	39	1	313	1	156	1
+Reserpine	156	2	19.5	2	313	1	156	1
+CCCP	156	2	19.5	2	313	1	156	1
**Syringic**	313		78		156		156	
+PAβN	156	2	<1.2	**>64**	19.5	**8**	<9.8	**>16**
+NMP	156	2	<1.2	**>64**	39	**4**	39	**4**
+Verapamil	313	1	39	2	78	2	156	1
+Reserpine	313	1	78	1	78	2	156	1
+CCCP	19.5	**16**	<1.2	**>64**	9.8	**16**	156	1
**Ferulic**	313		78		156		156	
+PAβN	<9.8	**>32**	<1.2	**>64**	39	**4**	<9.8	**>16**
+NMP	78	**4**	2.4	**32**	39	**4**	39	**4**
+Verapamil	156	2	78	1	313	0.5	156	1
+Reserpine	313	1	39	2	313	0.5	156	1
+CCCP	313	1	<1.2	**>64**	<9.8	**>16**	156	1

“**Fold diff.**” indicates fold difference, which is calculated using the formula: MIC without an EPI/MIC with an EPI. ≥4-fold changes are indicated in bold.

a PAβN and NMP significantly (*p*<0.05) reduced the MICs of the phenolic compounds in 11168, 11168B, 11168F, and 11168R.

b CCCP significantly reduced the MICs of the phenolic compounds in 11168, 11168B and 11168F (*p*<0.05), but not in 11168R (*p* > 0.05).

The antimicrobial activities against different *Campylobacter* strains are shown in Table 2, which showed variable anti-*Campylobacter* activities of the selected natural phenolic acids and plant extracts. The tested *C. coli* isolates (137, 140, 171, FC8, FC10, VC7114, VC10076) were previously shown to be resistant to erythromycin, ciprofloxacin and tetracycline [30]. Statistical analysis indicated no significant differences between erythromycin-susceptible and erythromycin-resistant isolates in their susceptibility to most of the examined pure phenolics and plant extracts. The statistical analysis also showed that most of the tested compounds had similar activities against both *C. coli* and *C. jejuni* isolates (Table 2). These results indicate that the tested phenolics and plant extracts are generally effective against both antibiotic-resistant and antibiotic-susceptible *Campylobacter* and suggest that the action mode of phenolic compounds is different from the antibiotics.

**Table 5:** Susceptibilities of *C. jejuni* 11168 and its efflux mutants to the selected plant extracts in the presence or absence of PAβN (20 µg/mL)^a^, NMP (100 µg/mL) a, verapamil (100 µg/mL), reserpine (100 µg/mL) or CCCP (0.25µg/mL)^b^.

Extract ±inhibitor	11168		11168B		11168F		11168R	
	MIC (µg/mL)	Fold diff.	MIC (µg/mL)	Fold diff.	MIC (µg/mL)	Fold diff.	MIC (µg/mL)	Fold diff.
**I18**	313		19.5		625		156	
+PAβN	<0.6	**>512**	19.5	1	39	**16**	39	**4**
+NMP	19.5	**16**	39	0.5	313	2	19.5	**8**
+Verapamil	39	**8**	19.5	1	625	1	156	1
+Reserpine	19.5	**16**	4.9	**4**	625	1	156	1
+CCCP	4.9	**64**	9.8	2	313	2	156	1
**V40**	78		9.8		156		156	
+PAβN	4.9	**16**	<0.3	**≥32**	9.8	**16**	9.8	**16**
+NMP	19.5	**4**	<4.9	≥2	78	2	78	2
+Verapamil	39	2	04.9	2	156	1	156	1
+Reserpine	39	2	9.8	1	78	2	78	2
+CCCP	4.9	**16**	9.8	1	78	2	156	1
**V70**	78		4.5		78		78	
+PAβN	19.5	**4**	9.8	0.5	<1.2	**≥64**	78	1
+NMP	39	2	9.8	0.5	19.5	2	39	2
+Verapamil	78	1	9.8	0.5	78	1	156	0.5
+Reserpine	78	1	9.8	0.5	78	1	156	0.5
+CCCP	39	2	4.5	1	39	2	78	1
**A40**	156		2.4		156		313	
+PAβN	39	**4**	0.3	**8**	78	2	39	**8**
+NMP	78	2	0.3	**8**	39	**4**	39	**8**
+Verapamil	156	1	1.2	2	313	0.5	313	1
+Reserpine	156	1	2.4	1	313	0.5	313	1
+CCCP	39	**4**	2.4	1	156	1	313	1
**Babić**	8,000		4,000		8,000		8,000	
+PAβN	2,000	**4**	250	**16**	500	**8**	500	**8**
+NMP	1,000	**8**	1,000	**4**	2,000	2	2,000	2
+Verapamil	8,000	1	2,000	2	4,000	1	2,000	2
+Reserpine	8,000	1	4,000	1	4,000	1	2,000	2
+CCCP	125	**64**	125	**32**	125	**32**	125	**32**

“**Fold diff.**” indicates fold difference, which is calculated using the formula: MIC without an EPI/MIC with an EPI. ≥4-fold changes are indicated in bold.

a PAβN and NMP significantly (*p*<0.05) reduced the MICs of the plant extracts in 11168, 11168F and 11168R, but not in 11168B (*p* > 0.05).

b The effect of CCCP on the MICs of the plant extracts was only significant with 11168 (*p*<0.05).

The MIC of NCTC 11168 and its mutants strains are shown in Table 3. Among the 9 pure phenolic compounds examined, the most effective ones were EGCG and carnosic acid, with a MIC of 78 µg/mL and 19.5 µg/mL, respectively, for wild-type 11168 (Table 3). Rosmarinic acid showed a good activity, too (MIC = 156 µg/mL). For the plant extracts, good antimicrobial activities were observed with rosemary extracts (V40, V70, A40), containing rosmarinic and carnosic acids as the major components. Additionally, phenolic extracts from peppermint and green tea showed activities similar to those detected for pure rosmarinic acid and the rosemary extract A40 (where rosmarinic acid is the main component). The other herb extracts (lemon balm, oregano, thyme, bearberry, black seeds) and some vine-leaf extracts (*Lasin, Merlot, Vranac, Debit, Zlatarica*) showed moderate anti-*Campylobacter* activities, with MICs from 313 µg/mL to 1,250 µg/mL. The other vine-leaf extracts (*Kujundžuša, Rkaciteli, Trnjak, Rudežuša,* and *Babić*) were less effective, with MICs of 1,000 µg/mL to 8,000 µg/mL (Table 3).

### Role of CmeABC and CmeDEF in the Resistance to the Natural Phenolic Compounds

We used gene knockout mutants to determine the specific roles of CmeABC and CmeDEF efflux pumps in the resistance to the natural phenolic compounds. The *cmeB* mutant (11168B), *cmeF* mutant (11168F) and *cmeR* mutant (11168R) were compared with the wild-type strain (11168) using the MIC assay. As shown in Table 3, the gene mutations had varied impacts on the susceptibility to the phenolic compounds and extracts. The insertional inactivation of *cmeB* resulted in the most obvious, statistically significant changes in the MICs and increased the susceptibility of *C. jejuni* NCTC 11168 to all but two of the tested compounds and extracts by 2-fold to 128-fold (Table 3), indicating that the CmeABC efflux pump plays an important and broad role in the resistance to phenolics. Notably, the MICs for rosmarinic, chlorogenic and gallic acids decreased 64- to 128-fold in 11168B compared with the wild-type strain, suggesting that CmeABC is especially effective in the efflux of these phenolic compounds. Similarly, significant increases in the susceptibilities in the *cmeB* mutant strain were seen for all of the rosemary extracts (8- to 64-fold), and for most of the herb (up to 128-fold) and vine-leaf (up to 64-fold) extracts. These data clearly indicate that these natural pure phenolic compounds and extracts of plant phenolics represent substrates for CmeABC in *C. jejuni*. Interestingly, inactivation of the CmeB efflux-pump protein did not affect the MICs of EGCG and carnosic acid (Table 3), suggesting that these two compounds are not the substrates of CmeABC. Alternatively, EGCG and carnosic acid may not enter into *Campylobacter* cells and act on membrane or cell surfaces [31], [32]. These two phenolics have the lowest MICs, confirming them as the most efficient anti-*Campylobacter* phenolics tested in this study.

In contrast to the results with 11168B, inactivation of the *cmeF* gene had much smaller effects (up to 8-fold reduction or 4-fold increase) on the MICs of these natural phenolic compounds (Table 3). The MICs for the pure gallic, sinapinic, syringic and ferulic acids and for most of the herb and vine-leaf extracts, were reduced by 2- to 8-fold. Interestingly, the *cmeF* inactivation increased the MICs of some of other compounds by 2- to 4-fold (e. g. rosmarinic acid, carnosic acid, rosemary extracts V40 and I18, bearberry, black seeds and grape leaf extract vranac) (Table 3). The data obtained here indicate that CmeDEF plays a modest role in modulating the resistance to different plant phenolic compounds in *C. jejuni*.

It is known from previous studies that CmeABC contributes to *Campylobacter* resistance to a broad spectrum of antimicrobial agents and is the predominant efflux system in *Campylobacter* [20]–[22], while CmeDEF plays a secondary role in conferring intrinsic resistance to antimicrobials [26]. Findings from this study are consistent with this notion as mutation of *cmeB* resulted in significantly greater changes in the MICs (Table 3). To our knowledge, this is the first study demonstrating that antibiotic efflux pumps extrude phenolic acids, compounds or phenolic extracts and contribute to the resistance of *C. jejuni* to these compounds. It is of particular interest that each pure phenolic compound or plant extract shows certain specificity for different efflux pumps, suggesting that structural variations of the phenolic compounds influence their interactions with the drug efflux transporters in *Campylobacter*. Based on the MIC differences observed with 11168 B and 11168F, we can conclude that CmeABC is the predominant efflux pump in *C. jejuni* for the efflux of pure phenolic compounds and phenolic extracts of plant origin.

CmeR functions as a transcriptional repressor that directly interacts with the *cmeABC* promoter and modulates the expression of *cmeABC* and mutation of *cmeR* will impede this repression, leading to enhanced production of the CmeABC MDR efflux pump [23]. As shown in Table 3, inactivation of *cmeR* indeed led to slightly increased (up to 4-fold) or reduced (4-fold) resistance to these natural phenolic compounds as reflected by the MIC changes in comparison with the wild-type strain. Four of these natural phenolic compounds (V70, peppermint, *Babić* and chlorogenic acid) did not show a change in MIC in 11168R. This *cmeR* inactivation resulted in a modest reduction in the MICs for most of the tested compounds and extracts. On the contrary, it increased the MICs of ECGC, rosmarinic and chlorogenic acid as well as some rosemary and vine-leaf extracts by up to 4-fold (Table 3). These results are consistent with a previous finding with other antimicrobials that overexpression of CmeABC (mediated by inactivating *cmeR*) only resulted in modest changes in drug resistance [23]. The small MIC changes in 11168R are in contrast to the significant MIC alterations in 11168B and suggest that the function of CmeABC is already saturated by the base-level expression and overexpression of this efflux pump does not further enhance its function in the extrusion of phenolic compounds. Alternatively, the modest changes of MICs in 11168R could be explained by the fact that CmeR regulates multiple genes in *C. jejuni* and inactivation of CmeR affects the expression (both down- and up-regulation) of a number of genes [33], which collectively might affect the impact of the *cmeR* mutation on the MICs.

### The Effects of EPIs on the Resistance to Natural Phenolic Compounds

In addition to using gene-specific mutants, we further examined the role of efflux mechanisms in the resistance to natural phenolic compounds using different EPIs including PAβN, NMP, verapamil, reserpine, and CCCP. Two (PAβN and NMP) of these EPIs have been evaluated to restore erythromycin susceptibility [24], [34]–[37] and influence the resistance to others antibiotics [30] in *Campylobacter* spp., but none of them has been tested to modulate the susceptibility of *Campylobacter* to phenolic acids or compounds of plant phenolic extracts.

In the present study, we examined the susceptibility of *C. jejuni* 11168 and its mutant constructs to 9 pure phenolic compounds and five phenolic extracts (four rosemary and vine-leaf extract) in the absence and presence of each EPI. The MIC values are given in Table 4 and Table 5. The resistance of *C. jejuni* 11168 to these natural phenolic compounds was significantly reduced by PAβN (from 2- to >512-fold MIC reductions), and the effects varied with different compounds (Tables 4 and 5). NMP and CCCP also produced variable but statistically significant decreases in the MICs. On the other hand, verapamil and reserpine had little or no effects on the MICs of these natural antimicrobials (Tables 4 and 5). These tested EPIs may have different modes of action in *Campylobacter*, thus showing highly divergent effects on the MICs of the tested phenolic compounds.

In 11168B, several EPIs increased its susceptibility to the pure phenolic compounds and extracts of plant phenolics by up to >64-fold. The MIC reduction was particularly obvious in the cases of carnosic, sinapinic, syringic and ferulic acids (Tables 4, 5). Similar to what was observed with the wild-type 11168, PAβN, NMP and CCCP showed greater, potentiating effects than the other EPIs (p<0.05). The fact that MICs in 11168B were further reduced by EPIs strongly suggests that other efflux mechanisms also contribute to *Campylobacter* resistance to natural phenolic compounds.

The EPIs were further evaluated in the *cmeF* mutant (11168F). Again, the significant potentiating effects (MIC reduction) were mainly seen with PAβN, NMP and CCCP, but the magnitudes of MIC reduction were generally smaller in 11168F than in 11168B and the wide-type strain, except for V70 and I18 rosemary, with which PAβN produced a greater MIC reduction in 11168F than in 11168B (Tables 4 and 5). In the *cmeR* mutant (11168R), PAβN significantly reduced the MICs for all of the pure phenolic compounds (with up to >128-fold MIC reductions), and for all of the extracts tested except V70. Interestingly, NMP produced a 256-fold reduction in the MIC of EGCG in 11168R, but had no or limited potentiating activity on EGCG in the wild-type and other mutant strains. This suggests that inactivation of CmeR might alter a mechanism in *C. jejuni*, which makes the organism significantly more susceptible to EGCG inhibition in the presence of NMP. For all of the tested pure phenolic compounds and plant extracts in the wild type and mutant strains (Tables 4 and 5), PAβN showed the most effective potentiating effects, followed by CCCP, NMP, reserpine and verapamil. Results from the EPI experiments further indicate the complexity of mechanisms that influence the susceptibility of *C. jejuni* to plant phenolic compounds.

This study represents a comprehensive evaluation of the anti-*Campylobacter* activities of natural phenolic compounds and extracts. All of the tested phenolics showed activities against *Campylobacter* spp. isolates from different sources, although their activities were variable and closely related to their compositions. Additionally, the tested natural phenolic compounds and plant extracts showed similar activities against both *C. jejuni* and *C. coli* as well as antibiotic resistant *Campylobacter*, suggesting that they may be potentially used as alternative antimicrobials for the control of sensitive and multidrug-resistant *Campylobacter*. Although practical use of these plant compounds requires further research and development, it is possible that they can be developed for use in live birds or processed meat to reduce *Campylobacter* colonization and contamination. Poultry are a major reservoir for *Campylobacter* and contaminated poultry meat serves as a major vehicle for foodborne transmission of *Campylobacter* humans [1]. Due to the rising prevalence of antibiotic resistance, alternatives to traditional antibiotics are needed to control *Campylobacter* in animal reservoirs. One potential use of these plant compounds could be incorporated into feed or water to reduce the colonization and prevalence of *Campylobacter* in birds at the preharvest stage. Additionally, the natural plant antimicrobials may be used as additives, preservation or decontamination treatments to reduce *Campylobacter* contamination on chicken carcasses during the post-harvest stage.

To facilitate the practical use of these phenolics, it is important to understand the factors in *C. jejuni* that affect the susceptibility to the antimicrobials. Using gene-specific knockout mutants and EPIs, we demonstrated that complex efflux mechanisms are involved in the resistance of *C. jejuni* to phenolic compounds and extracts of plant phenolics (Tables 3, 4 and 5). Particularly, the CmeABC efflux pump is a significant player in reducing the susceptibility to the phenolics, while CmeDEF plays a modest role in the resistance. Additionally, our results suggest that non-CmeABC and non-CmeDEF efflux systems also contribute to *Campylobacter* resistance to phenolic compounds. Collectively, these findings represent the first comprehensive evaluation of the anti-*Campylobacter* activities of plant phenolic compounds and suggest that these compounds can be further developed as alternative antimicrobials to control *Campylobacter* contamination in food production and processing, or as therapeutics for clinical treatment of campylobacteriosis. These possibilities await investigations in future studies.
